# Development of fully defined xeno-free culture system for the preparation and propagation of cell therapy-compliant human adipose stem cells

**DOI:** 10.1186/scrt175

**Published:** 2013-03-07

**Authors:** Mimmi Patrikoski, Miia Juntunen, Shayne Boucher, Andrew Campbell, Mohan C Vemuri, Bettina Mannerström, Susanna Miettinen

**Affiliations:** 1 Adult Stem Cell Group, Institute of Biomedical Technology, University of Tampere, Tampere, Finland; 2 BioMediTech, Tampere, Finland; 3 Science Center, Tampere University Hospital, Tampere, Finland; 4 Life Technologies, Cell Therapy Systems, Frederick, MD, USA; 5 Life Technologies, BioProduction, Grand Island, MD, USA

**Keywords:** Adipose stem cells, Xeno-free, Serum-free, Human serum, Fetal bovine serum, Multipotentiality, Proliferation rate, Immunophenotype, Flow cytometry, Cell therapy

## Abstract

**Introduction:**

Adipose tissue is an attractive and abundant source of multipotent stem cells. Human adipose stem cells (ASCs) have shown to have therapeutic relevancy in diverse clinical applications. Nevertheless, expansion of ASCs is often necessary before performing clinical studies. Standard *in vitro* cell-culture techniques use animal-derived reagents that should be avoided in clinical use because of safety issues. Therefore, xeno- and serum-free (XF/SF) reagents are highly desirable for enhancing the safety and quality of the transplanted ASCs.

**Methods:**

In the current study, animal component-free isolation and cell-expansion protocols were developed for ASCs. StemPro MSC SFM XF medium with either CELLstart™ CTS™ coating or Coating Matrix Kit were tested for their ability to support XF/SF growth. Basic stem-cell characteristics such as immunophenotype (CD3, CD11a, CD14, CD19, CD34, CD45RO, CD54, CD73, CD80, CD86, CD90, CD105, HLA-DR), proliferation, and differentiation potential were assessed in XF/SF conditions and compared with human serum (HS) or traditionally used fetal bovine serum (FBS) cultures.

**Results:**

ASCs cultured in XF/SF conditions had significantly higher proliferation rates compared with HS/FBS cultures. Characteristic immunophenotypes of ASCs were maintained in every condition; however, cells expanded in XF/SF conditions showed significantly lower expression of CD54 (intercellular adhesion molecule 1, ICAM-1) at low passage number. Further, multilineage differentiation potential of ASCs was maintained in every culture condition.

**Conclusions:**

Our findings demonstrated that the novel XF/SF conditions maintained the basic stem cell features of ASCs and the animal-free workflow followed in this study has great potential in clinical cell therapies.

## Introduction

Human adipose tissue is an abundant source of multipotent stem cells known as adipose stem cells (ASCs), and they have the ability to differentiate toward various mesenchymal cell types, including bone, cartilage, and fat cells [1,2]. Since Zuk *et al.*[3] described this unlimited source of multipotent cells, growing interest exists toward the clinical applicability of ASCs. The therapeutic relevance of the cells has been noticed and, in fact, the number of clinical cell therapies using ASCs has been steadily increassing [4-6]. Therefore, increased focus occurs on the safety, efficacy, reproducibility, and quality of the cells used in clinical treatments.

ASCs and bone marrow-derived stem cells (BMSCs) alike are mesenchymal stem cells (MSCs) that are defined as plastic-adherent cells with the potential to differentiate toward bone-, fat-, and cartilage-like cells. Furthermore, for their characterization, it is required that the cells express (≥95%) certain markers on the cell surface (CD105, CD73, and CD90) and lack the expression of hematopoietic antigens (≤2%) [7].

Of particular interest is that ASCs have immunomodulatory properties such as regulation of T-cell functions, antiinflammatory cytokine expression, and prolongation of allotransplant survival [8,9]. Thus, ASCs have been shown to suppress allogeneic lymphocytes both *in vitro* and *in vivo*[9,10]. In addition, ASCs lack the expression of MHC class II molecules as well as T- and B-cell co-stimulatory molecules CD80, CD86, and CD40 on their cell surfaces [11]. Because of these characteristics, ASCs are strong candidates for the treatment of immunologic disorders such as severe graft-versus-host disease or Crohn disease. Furthermore, because of their low immunogenicity, they may be used in allogeneic stem cell therapies, such as in the treatment of bone defects. High proliferation rate and good differentiation potential are important from the clinical point of view, and therefore, off-the-shelf cell products could be used to achieve effective treatments by using allogeneic cells with functional stem cell characteristics.

Cell-based therapies typically require large numbers of cells, and expansion of ASCs is often necessary before clinical use. Traditionally, fetal bovine serum (FBS) has been used in ASC cultures because of its ability to support cell growth and attachment by providing nutrients and attachment factors for the cells. However, in clinical cell therapies, the use of animal-derived reagents should be avoided, and the risks and benefits carefully assessed because of safety concerns [12,13]. Alternatives for FBS have been studied, such as allogeneic human serum (alloHS) or autologous HS (autoHS) [14], as well as platelet-derived supplements [15,16]. Nevertheless, limitations connected to the use of serum or its components exist, such as lot-to-lot variation with serum-supplemented media performance, affecting differentiation potential and proliferation rate [17]. In addition, limited availability of autoHS can make long-term cultures of ASCs impractical. It is noteworthy that no standardized, fully defined xeno- and serum-free (XF/SF) cultivation protocols are available. However, the safety and the quality of transplanted ASCs would be significantly enhanced by replacing undefined and animal-derived components with defined XF/SF reagents.

The aim of the current study was to develop safe and efficient XF/SF culture conditions for ASCs and to show that ASCs cultured under these novel XF/SF conditions maintained their stem-cell characteristics, including the multilineage differentiation potential, immunophenotype, and proliferation capacity. The cell isolation and expansion was carried out in parallel in three different culture conditions, under fully defined completely XF/SF conditions, as well as in medium containing HS or FBS to compare the cell characteristics between these conditions. This study demonstrates the development of a fully defined animal origin-free culture system for the propagation and expansion of clinically relevant human adipose stem cells for the purpose of cell therapy.

## Methods

### Isolation and culture of ASCs

The study was conducted in accordance with the ethics committee of the Pirkanmaa Hospital District, Tampere, Finland (R03058). ASCs were isolated from adipose tissue samples obtained with written informed consent from four female donors (age, 36 ± 9 years) undergoing elective surgical procedures in the Department of Plastic Surgery, Tampere University Hospital, Tampere, Finland. To assess how serum supplementation of the culture media affects the cell characteristics, ASCs were isolated under three different culturing conditions: in medium containing FBS, HS, or in XF/SF culture conditions. FBS- and HS-containing media were used as reference media for testing two different XF/SF culture conditions: (a) XF/SF media with CELLStart coating, and (b) XF/SF media with novel coating-free supplement, referred to hereafter as Coating Matrix Kit.

Isolation of adipose stem cells (ASCs) from adipose tissue samples was carried out by using a mechanical and enzymatic method, as described previously [1,18,19]. In brief, the adipose tissue was minced manually into small fragments and digested with collagenase NB 6 GMP Grade (SERVA Electrophoresis GmbH, Heidelberg, Germany) in a water bath at 37°C under shaking conditions. The digested tissue was centrifuged and filtered in sequential steps through a 100-μm pore-size filter to separate the ASCs from the surrounding tissue. The first passage after the seeding of cells on cell-culture plastics, after dissociation of fat tissue, was designated passage 0. Cells were expanded in T75 flasks and passaged after reaching 80% confluency.

For HS and FBS conditions, Dulbecco modified Eagle medium (DMEM)/F-12 1:1 (Life Technologies, Gibco, Carlsbad, CA, USA) was supplemented with 1% l-analyl-l-glutamine (GlutaMAX I; Life Technologies, Gibco), 1% antibiotics (p/s; 100 U/ml penicillin, 0.1 mg/ml streptomycin; Lonza, BioWittaker, Verviers, Belgium) and serum from either 10% FBS (Life Technologies, Gibco) or 10% alloHS (Human Serum Type AB; Lonza, BioWhittaker, Walkersville, MD, USA) was used. ASCs isolated and expanded in FBS medium were detached by using 1% trypsin (Lonza, Biowhittaker, Verviers, Belgium), and ASCs isolated in HS medium were detached by using TrypLE Select (Life Technologies, Gibco) for XF detachment of cells.

For SF/XF conditions, one third of the cells were isolated under SF/XF conditions, and seeded on carboxyl-coated flasks (PureCoatCarboxyl T75; BD Biosciences, Franklin Lakes, NJ, USA) and expanded in STEMPRO® MSC SFM (Life Technologies, Gibco) supplemented with 1% l-analyl-l-glutamine, 0.3% antibiotics, and 10% StemPro MSC SFM XenoFree supplement. Amine-coated flasks (PureCoat™ Amine T75; BD Biosciences) were initially tested for their suitability for XF/SF, but the coating was not supportive enough for cell attachment, and instead of amine flasks, the carboxyl-coated flasks were selected for further studies.

From passage 1 onward, additional supplements were used in XF/SF conditions to support cell attachment and growth in normal Nunclon flasks. Thus, XF/SF cells were expanded in STEMPRO MSC medium supplemented with either Coating Matrix Kit (XF/SF CM) (Life Technologies, Gibco) or CELLstart™ CTS™ coating (XF/SF CS) (Life Technologies, Gibco), according to manufacturer's instructions. ASCs isolated and expanded in SF/XF medium were detached by using TrypLE Select for XF detachment of cells.

**Figure 1:**
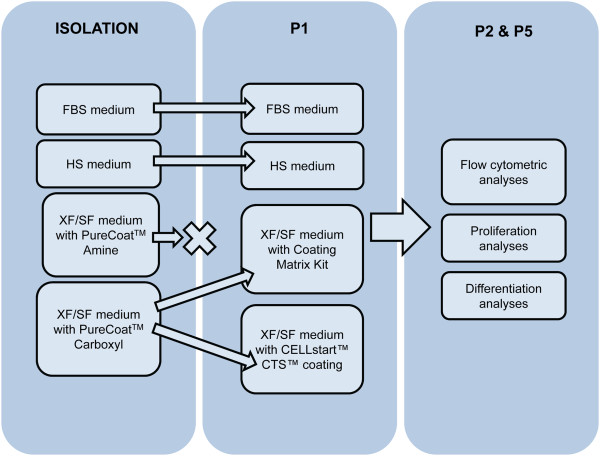
**Work flow of the isolation and performed analyses of ASCs under different culture conditions.** XF/SF isolation of ASCs was carried out by using carboxyl-coated flasks, and onward from passage 1, two different XF/SF conditions were tested in basic Nunclon flasks; Coating Matrix Kit, and CELLstart™ coating. Cell-proliferation rate, differentiation potential, and immunophenotype were analyzed in four different culture conditions at passages 2 and 5.

All culture-media formulations are presented in Table 1, and a flow chart of the isolation as well as performed analyses in different culture conditions are illustrated in Figure 1. All the analyses were performed separately with four donor cell lines isolated in FBS, HS, and XF/SF conditions.

**Table 1:** Culture-media formulation overview

**Acronym**	**Basal media**	**Serum**	**Coating/coating-free supplements**	**Supplementation**
HS	DMEM/F-12	Human serum	None	1% GlutaMAX, 1% p/s
FBS	DMEM/F-12	Fetal bovine serum	None	1% GlutaMAX, 1% p/s
XF/SF CM	StemPro MSC SFM	None	Coating Matrix Kit	StemPro® MSC SFM XenoFree supplement,
				1% GlutaMAX, 0.3% p/s
XF/SF CS	StemPro MSC SFM	None	CELLstart™ coating	StemPro MSC SFM XenoFree supplement,
				1% GlutaMAX, 0.3% p/s

MSC, mesenchymal stem cell; p/s, penicillin/streptomycin; SFM, serum-free medium.

### Proliferation assay

The cell viability and proliferation activity were assessed in the different culture conditions (FBS, HS, and SF/XF) by using the PreMix WST-1 Cell Proliferation Assay System (Takara Bio Inc., Shiga, Japan). ASCs (*n* = four donor cell samples/analysis, passages 2 and 5) were seeded on 48-well plates at a density of 2,500 cells/cm^2^, and the proliferation was assessed at 1, 4, 7, and 11 days. In brief, at each time point, the cell-culture medium was removed, and DPBS (Dulbecco Phosphate-Buffered Saline, Lonza, BioWhittaker, Verviers, Belgium) and PreMix WST-1 were added 10:1. The 48-well plate was incubated for 4 hours at 37°C, and the relative cell-proliferation activity was measured in a microplate reader (Victor 1429 Multilabel Counter) at 450 nm.

The population doubling was determined by using the formula x = log2(NH)/(N1), where *N*1 is the absorbance value at day 1, and *N*H is the absorbance value at observed time point 4, 7, or 11, as described previously [20]. To calculate the cumulative population doubling, the population doubling was determined in each passage and compared with the population doubling of earlier passages.

### Flow-cytometric analysis of immunophenotype

ASCs expanded in SF/XF, HS, and FBS (*n* = 4, passages 2 and 5) media were analyzed with flow cytometry (FACSAria; BD Biosciences, Erembodegem, Belgium) to determine whether different culturing conditions have an effect on the immunophenotype of the cells. Monoclonal antibodies (MAbs) against CD11a–allophycocyanin (APC), CD80–phycoerythrin (PE), CD86–PE, CD105–PE (R&D Systems Inc., Minneapolis, MN, USA), CD-3 (PE), CD14–phycoerythrin-cyanine (PECy7), CD19-PECy7, CD45RO-APC, CD54-fluorescein isothiocyanate (FITC), CD73-PE, CD90-APC (BD Biosciences), and CD34-APC, HLADR-PE (Immunotools GmbH, Friesoythe, Germany) were used. Analysis was performed on 10,000 cells per sample, and unstained cell samples were used to compensate for the background autofluorescence levels.

### Differentiation analyses

The trilineage differentiation potential of ASCs (*n* = 4, passages 2 to 5) toward osteogenic, adipogenic and chondrogenic cells was evaluated in XF/SF conditions versus HS and traditionally used FBS-supplemented medium. Differentiation capacity of ASCs was evaluated after 14 days of differentiation in either adipogenic, osteogenic, or chondrogenic medium versus cells cultured in control medium. Media for differentiation and control cultures were changed 3 times per week during the differentiation studies. The culture-media formulations used for differentiation assays are shown in Table 2. In a subsequent smaller-scale study, ASCs were primed for 3 days under FBS- or HS-supplemented media before differentiating under osteogenic or adipogenic condition. For this, commercial serum-based StemPro Adipogenesis and Osteogenesis differentiation kits (Life Technologies, Gibco) were used during the 14-day induction for XF/SF cells.

**Table 2:** Culture media formulations used for differentiation assays

**Medium**	**Basal media**	**Serum**	**Coating/coating-free supplements**	**Supplementation**
Adipogenic (FBS)	DMEM/F-12	10% Fetal bovine serum	None	1% GlutaMAX, 1% p/s, 33 μ*M* biotin (Sigma), 1 μ*M* dexamethasone (Sigma), 100 n*M* insulin (Life Technologies), 17 μM pantothenate (Fluka, Buchs, Switzerland), 250 μ*M* isobutylmethylxanthine (IBMX; Sigma) for 48-hour induction after cell seeding
Osteogenic (FBS)	DMEM/F-12	10% Fetal bovine serum	None	1% GlutaMAX, 1% p/s, 150 μ*M* L-ascorbic acid 2-phosphate (Sigma), 10 m*M* β-glycerophosphate (Sigma), 10 n*M* dexamethasone (Sigma)
Chondrogenic (FBS/HS)	DMEM/F-12	None	None	1% GlutaMAX, 0.3% p/s, 10 mg/ml human serum albumin (Sigma), 8 μg/ml holo-transferrin human (Sigma), 5 ng/ml sodium selenite (Sigma), 10 μg/ml insulin (Life Technologies), 1 μg/ml linoleic acid (Sigma), 50 μ*M* L-ascorbic acid 2-phosphate (Sigma), 55 μ*M* sodium pyruvate (Life Technologies), 23 μ*M* L-proline (Sigma), 10 ng/ml TGF-β (Sigma)
Adipogenic (HS)	DMEM/F-12	10% Human serum	None	1% GlutaMAX, 1% p/s, 33 μ*M* biotin, 1 μ*M* dexamethasone, 100 n*M* insulin, 17 μ*M* pantothenate, 250 μ*M* IBMX for 48-hour induction after cell seeding
Osteogenic (HS)	DMEM/F-12	10% Human serum	None	1% GlutaMAX, 1% p/s, 150 μ*M* L-ascorbic acid 2-phosphate, 10 m*M* β-glycerophosphate, 10 n*M* dexamethasone
Adipogenic (XF/SF CS)	StemPro MSC	none	CELLstart™ coating	StemPro MSC SFM XenoFree supplement, 1% GlutaMAX, 0.3% p/s, 33 μ*M* biotin, 1 μ*M* dexamethasone, 100 n*M* insulin, 17 μ*M* pantothenate, 250 μ*M* IBMX for 48-hour induction after cell seeding
	SFM			
Osteogenic (XF/SF CS)	StemPro MSC	none	CELLstart™ coating	StemPro MSC SFM XenoFree supplement, 1% GlutaMAX, 0.3% p/s, 150 μ*M* L-ascorbic acid 2-phosphate, 10 m*M* β-glycerophosphate, 10 n*M* dexamethasone
	SFM			
Chondrogenic (XF/SF CS and XF/SF CM)	StemPro MSC	None	None	1% GlutaMAX, 0.3% p/s, 10 mg/ml human serum albumin, 8 μg/ml holo-transferrin human, 5 ng/ml sodium selenite, 10 μg/ml insulin, 1 μg/ml linoleic acid, 50 μ*M* L-ascorbic acid 2-phosphate, 55 μ*M* sodium pyruvate, 23 μ*M* L-proline, 10 ng/ml TGF-β
	SFM			
Adipogenic (XF/SF CM)	StemPro MSC	None	Coating Matrix Kit	StemPro MSC SFM XenoFree supplement, 1% GlutaMAX, 0.3% p/s, 33 μ*M* biotin, 1 μ*M* dexamethasone, 100 n*M* insulin, 17 μ*M* pantothenate, 250 μ*M* IBMX for 48-hour induction after cell seeding
	SFM			
Osteogenic (XF/SF CM)	StemPro MSC	None	Coating Matrix Kit	StemPro MSC SFM XenoFree supplement, 1% GlutaMAX, 0.3% p/s, 150 μM L-ascorbic acid 2-phosphate, 10 mM beta-glycerophosphate, 10 nM dexamethasone
	SFM			

### ALP staining

For alkaline phosphatase (ALP) staining, cells were seeded on 12-well plates at a density of 2.5 × 10^3^ cells/cm^2^. The differentiation degree after 14 days of osteogenic induction was determined by the level of ALP activity by using a leukocyte ALP kit (Sigma-Aldrich, St. Louis, MO, USA), as described previously [21]. In brief, cell cultures were washed twice with DPBS and fixed with 4% paraformaldehyde (PFA) or citrate-buffered formaldehyde-acetone solution. Subsequently, cells were rinsed with deionized water, and ALP staining solution was added and incubated for 15 minutes. After rinsing the cells with deionized water, color formation was analyzed microscopically.

### Oil Red-O staining

For adipogenic differentiation, ASCs were seeded on 12-well plates at a density of 2.0 × 10^4^ cells/cm^2^. After 14 days of adipogenic induction culture, differentiation was confirmed by Oil Red-O staining, indicating the formation of intracellular lipid accumulation, as described earlier [2]. In brief, the cells were washed 3 times in DPBS and fixed with 4% PFA. Subsequently, cells were rinsed with deionized water and pretreated with 60% isopropanol after the additions of the 0.5% Oil Red-O staining solution in 60% isopropanol (Sigma-Aldrich). After 15-minute incubation in RT, the cells were rinsed with deionized water, and adipocytes were identified with microscopy as cells with red-stained lipid vesicles. In a later study, the cells were directly fixed and stained with 0.5% Oil Red-O staining solution with 60% isopropanol and then rinsed with distilled water before conducting microscopic assessment of adipocyte generation.

### Alcian blue staining

The chondrogenic differentiation potential was assessed with a micromass culture method, as described previously [3,19,22]. In brief, 8 × 10^4^ cells were seeded on a 24-well culture plate in a 10-μl volume and were allowed to adhere for 3 hours before the addition of chondrogenic induction medium. After 14 days of chondrogenic induction, differentiation was confirmed by using the Alcian blue staining method, as described earlier [23]. In brief, ASC pellets were rinsed with DPBS and fixed with 4% PFA. Subsequently, cells were rinsed twice with deionized water and stored in 70% ethanol. Pellets were dehydrated, embedded in paraffin, and sectioned at 5-mm thickness. The sections were rehydrated and stained with Alcian blue (pH 1.0) to detect sulfated glycosaminoglycans (GAGs) by using Nuclear Fast Red solution (Biocare Medical, Concord, MA, USA) as a counterstain.

### Real-time quantitative PCR

Total RNA was isolated by using the NucleoSpin RNA II kit (Macherey-Nagel, Düren, Germany) according to manufacturer’s instructions. The RNA samples were reverse transcribed to first-strand cDNA by using the High-Capacity cDNA Reverse Transcriptase Kit (Applied Biosystems, Foster City, CA, USA). The mRNA levels of adipogenesis/osteogenesis-associated genes were analyzed by the qRT–PCR method as described previously [23]. In brief, the real-time detection of PCR product was monitored by using the SYBR Green dye (Applied Biosystems, Warrington, UK). The housekeeping gene, the ribosomal phosphoprotein P0 (*RPLP0*), was used as an internal control, and the relative expression level for each gene was calculated according to a previously described mathematical model [24]. The expression of adipogenesis-associated genes, peroxisome proliferator-activated receptor γ (*PPARγ*), and adipocyte Protein 2 (*aP2*) was analyzed as well as osteogenesis-associated genes such as distal-less homeobox transcription factor 5 (*DLX5*), *ALP*, and runt-related transcription factor 2 (*RUNX2*). Sequences and accession numbers of all primers (Oligomer Oy, Helsinki, Finland) are displayed in Table 3. The reactions were conducted and monitored with ABI Prism 7000 Sequence Detection System (Applied Biosystems, Warrington, UK).

**Table 3:** Primer sequences of marker genes determined

**Name**	**Primer direction**	**Sequences**	**Product size (bp)**
*hRPLP0*^1^	Frw	5^′^-AAT CTC CAG GGG CAC CAT T-3^′^	70
	Rev	5^′^-CGC TGG CTC CCA CTT TGT-3^′^	
*haP2*^2^	Frw	5^′^-GGTGGTGGAATGCGTCATG-3^′^	71
	Rev	5^′^-CAACGTCCCTTGGCTTATGC-3^′^	
*hPPARG*^3^	Frw	5^′^-CAGTGTGAATTACAGCAAACC −3^′^	100
	Rev	5^′^-ACAGTGTATCAGTGAAGGAAT-3^′^	
*hRUNX2*^4^	Frw	5^′^-CCCGTGGCCTTCAAGGT-3^′^	76
	Rev	5^′^-CGTTACCCGCCATGACAGTA-3^′^	
*hDLX5*^5^	Frw	5^′^-ACCATCCGTCTCAGGAATCG-3^′^	75
	Rev	5^′^-CCCCCGTAGGGCTGTAGTAGT-3^′^	
*hALP*^6^	Frw	5^′^-ATGTCATCATGTTCCTGGGAGAT-3^′^	79
	Rev	5^′^-TGGTGGAGCTGACCCTTGAG-3^′^	

^1^Ribosomal protein, large, P0, (Acc. No: NM_001002); ^2^fatty acid-binding protein 4, (Acc. No: NM_001442); ^3^peroxisome proliferator-activated receptor gamma (Acc. No: NM_015869), ^4^runt-related transcription factor 2 (Acc. No: NM_004348); ^4^distal-less homeobox transcription factor 5 (Acc. No: NM_005221); ^5^alkaline phosphatase (Acc. No: NM_000478).

### Statistical analyses

One-way ANOVA with Bonferroni *post hoc* test was used to analyze the effect of different culture conditions on cell-proliferation rate, cell surface-marker expression, and differentiation potential by using IBM SPSS software version 19 (IBM SPSS Statistics 19, USA). Differences in proliferation rate between different culture conditions were analyzed separately at each time point. The statistical analyses were performed at the significance level *P* < 0.05, and data are presented as mean ± SD.

## Results

### XF/SF isolation of ASCs was the most critical step of the cell culture

**Figure 2:**
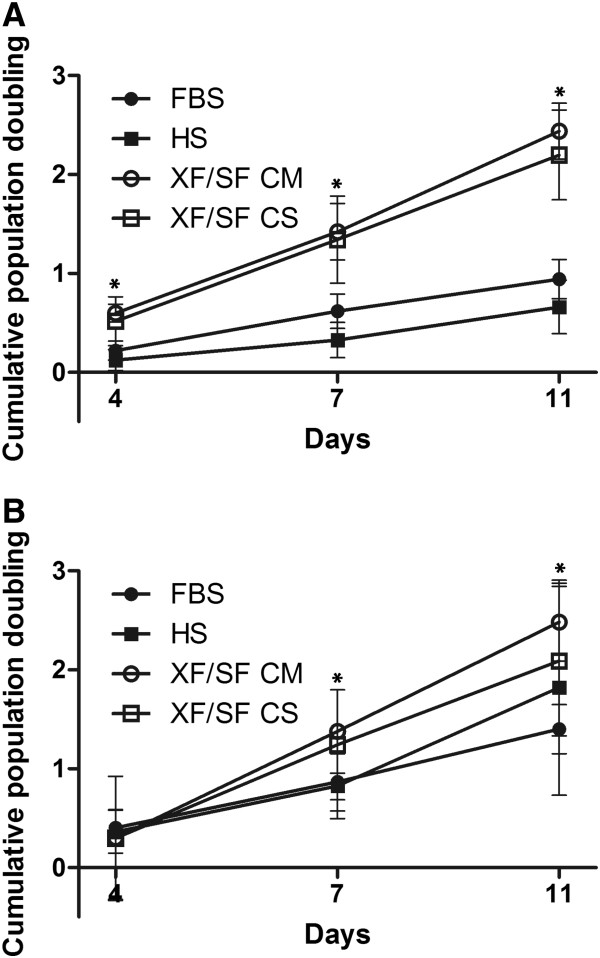
**WST-1 proliferation assay.** Cumulative population doubling was analyzed in different culture conditions, in FBS- and HS-containing medium, as well as in XF/SF medium with Coating Matrix Kit and with CELLstart™ coating at time points 1, 4, 7, and 11 day in two passages 2 (**A**) and 5 (**B**). The data in the diagrams are presented as mean ± SD. Significantly higher cumulative population doubling was observed in XF/SF conditions compared with HS/FBS cultures in passage 2 at 4-, 7-, and 11-day time points. Furthermore, statistically significant difference in population doubling were seen in passage 5 at 7- and 11-day time points between cells grown in XF/SF CM medium and FBS-containing medium.

The isolation of ASCs was conducted in three different culture conditions, in completely XF/SF conditions by using carboxyl-coated flasks (PureCoat™, BD), as well as in HS- and FBS-supplemented medium by using normal Nunclon™ cell-culture flasks. Carboxyl coating was used during the passage 0 after isolation in XF/SF conditions because Nunclon™ cell-culture flasks were unable to provide sufficient initial cell adhesion for ASCs in XF/SF medium. Still, after the first passaging, ASCs were able to grow in normal Nunclon™ flasks in XF/SF medium in the presence of Coating Matrix kit or CELLstart™ coating. The adhesion of ASCs after isolation in XF/SF medium was a critical step of XF/SF culture, and the XF/SF isolation was not successful with all the cell lines; cells from six donors were isolated, but only four donor cell lines were able to adhere and stay viable under XF/SF conditions. Thus, the isolation efficiency in XF/SF conditions was donor dependent. Nevertheless, if the cells were initially able to adhere, cell proliferation in XF/SF medium was efficient in further passages, and the cell-population doubling was notably faster than that in FBS/HS-containing medium (Figure 2). Subsequently, all experiments were carried out with four donor cell lines isolated in HS, FBS, and XF/SF conditions.

### Cumulative population doubling of ASCs expanded under XF/SF conditions versus medium containing HS or FBS

The cumulative population doubling of ASCs in XF/SF medium versus serum-containing medium was analyzed with WST-1 assay at time points 1, 4, 7, and 11 days in two passages, 2 and 5. A statistically significant increase in population doubling was seen in cells grown in XF/SF conditions compared with serum containing medium in passage 2 at 4-, 7-, and 11-day time points (Figure 2). Furthermore, a statistically significant difference was seen in passage 5 at 7- and 11-day time points between cells grown in XF/SF CM medium and FBS-containing medium (Figure 2).

Differences in population doublings between passages 2 and 5 were also statistically significant. In HS-supplemented medium; population doubling in passage 5 was significantly increased as compared with passage 2 at 4-, 7-, and 11-day time points, and in FBS medium, a statistically significant increase was seen in passage 5 at days 4 and 11 time points (Figure 2). Of note in passage 5, the population doubling in HS medium was higher than in FBS-containing medium at 11 days, whereas in passage 2, it was vice versa.

### Morphology of ASCs expanded under XF/SF conditions versus HS- or FBS-containing medium

**Figure 3:**
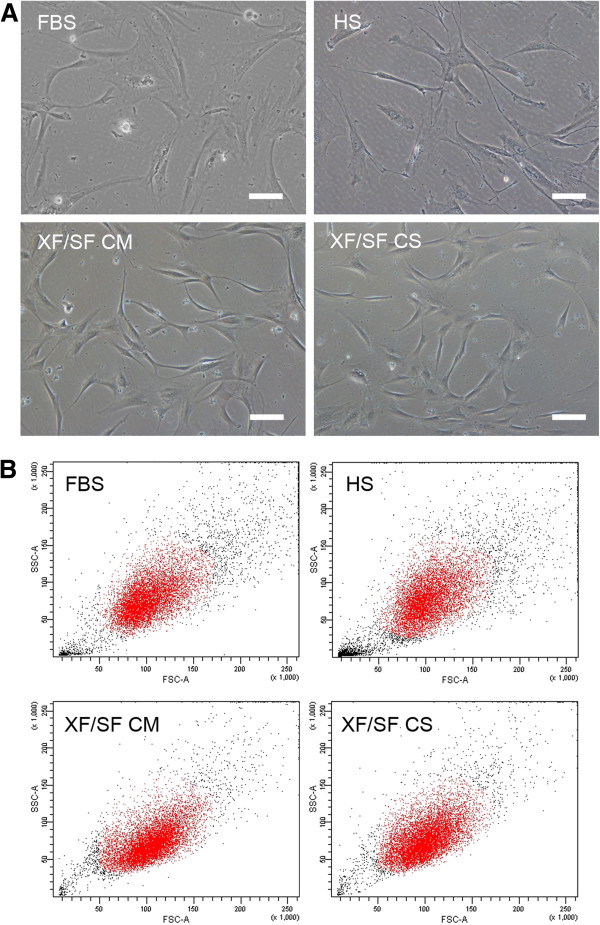
**Cell morphology. **(**A**) Morphologic images of cells cultured in different conditions: FBS, HS, XF/SF CM, and XF/SF CS at time point 4 days in passage 2. The morphology of ASCs grown in XF/SF medium is more spindle-shaped and smaller than in cells grown under serum-containing medium (FBS, HS). Scale bar, 100 μm. (**B**) Flow-cytometric analysis confirms the morphologic characteristics observed with light microscopy. In XF/SF culture conditions (CM and CS), the cell cloud in the forward and side scatter is more uniform and contains less debris than does a cloud of cells grown in FBS or HS medium, suggesting a more homogeneous population.

The morphologic differences between cells cultured in different conditions were consistent with the cell characteristics seen during the proliferation experiments. The adhesion of the cells grown in XF/SF medium was relatively weak during the isolation, which was also reflected in the morphology of ASCs in XF/SF conditions. Cells grown in the presence of serum adopted wide spindle-shaped and almost cuboidal morphology, whereas XF/SF cells were smaller, more spindle-shaped, and more fibroblast like (Figure 3). The morphologic differences suggest that spindle-shaped cells may not be as strongly attached as cells grown in serum-containing medium.

In addition to light microscopy, cell populations were examined by flow cytometry, especially the uniformity of the cells in the forward and side scatter. The cells expanded in XF/SF medium displayed a more homogeneous population, seen as a uniform cluster with less debris when compared with cells expanded in serum-containing medium (Figure 3).

### Immunophenotype of ASCs expanded under XF/SF conditions versus HS- or FBS-containing medium

**Figure 4:**
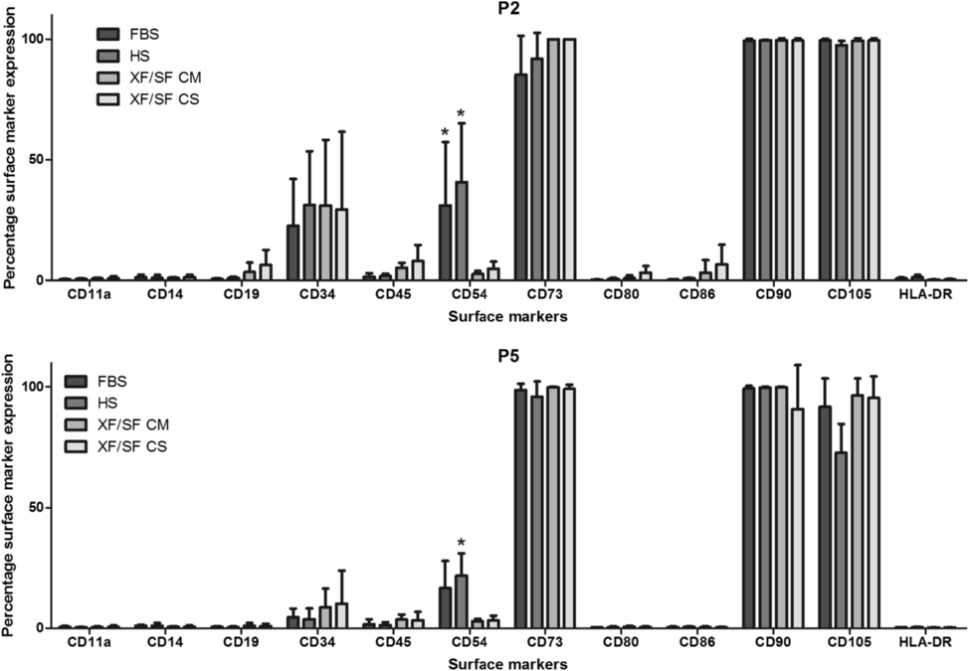
**Surface-marker expression of undifferentiated ASCs.** Immunophenotype of cells expanded in four different culture conditions; FBS, HS, XF/SF CM, and XF/SF CS was investigated in passages 2 and 5. The data in the diagrams are presented as mean ± SD. Cells expanded under XF/SF conditions showed significantly lower expression of CD54 (ICAM-1) compared with cells expanded in serum containing medium in passage 2. Furthermore, statistically significant differences in the expression of CD54 were seen between HS medium and XF/SF conditions in passage 5. Characteristic immunophenotypes of ASCs were maintained in every culture conditions with minor differences.

Cell-surface marker expression of ASCs was analyzed with flow cytometry to compare the expression profile of cells expanded in XF/SF conditions against cells expanded in HS- or FBS-containing medium at passages 2 and 5 (Figure 4). In general, the characteristic immunophenotype of ASCs was maintained in every culture condition, with some minor differences observed between XF/SF conditions and serum-containing media, as well as in the expression of specific markers between passages 2 and 5.

ASCs showed positive expression (>90%) for the markers CD73 (Ecto 5’ nucleotidase), CD90 (Thy-1) and CD105 (Endoglin) in all of the studied culture conditions in both passages (Figure 4), except the slightly lower expression of CD73 in FBS medium in P2 and CD105 in HS medium in P5. In contrast, ASCs lacked the expression (<2%) of CD11a (Integrin α-L), CD14 (LPS-Receptor), CD19 (B4), CD80 (B7-1), CD86 (B7-2), and HLA-DR (major histocompatibility class II receptor) in every culture condition with a few exceptions. Low expression (>2% to <7%) was observed for cells grown in XF/SF CM condition at P2 (CD19, CD86), and in XF/SF CS condition at P2 (CD19, CD80, CD86).

Moderate expression (>7% to <41%) was observed for the hematopoietic progenitor and endothelial cell marker CD34, except for the low expression in FBS and HS cultures at P5. ASCs lacked the expression of leukocyte common antigen CD45 in FBS and HS cultures, and low expression was observed in XF/SF conditions. The largest variation between different culture conditions was seen in the expression of CD54, which showed significantly lower expression in cells expanded under XF/SF conditions compared with cells expanded in serum-containing medium at P2. Furthermore, statistically significant differences were seen between HS medium and XF/SF conditions at passage 5. Generally, ASCs cultured in FBS or HS medium showed moderate expression of CD54 (intercellular adhesion molecule 1, ICAM1), whereas low expression was observed for cells cultured in XF/SF conditions. In addition, whereas the expression of CD34 and CD54 was decreased from passage 2 to passage 5, no statistical differences were observed between passages.

### Multipotentiality of ASCs expanded under XF/SF conditions versus HS- or FBS-containing medium

To test the multilineage differentiation potential of ASCs expanded under XF/SF conditions versus HS or FBS medium, the differentiation capacity toward the adipogenic, osteogenic, and chondrogenic lineages was analyzed. After the 14 days of differentiation induction, the differentiation degree was examined by specific staining methods and by the analysis of gene expression.

### Adipogenic differentiation

**Figure 5:**
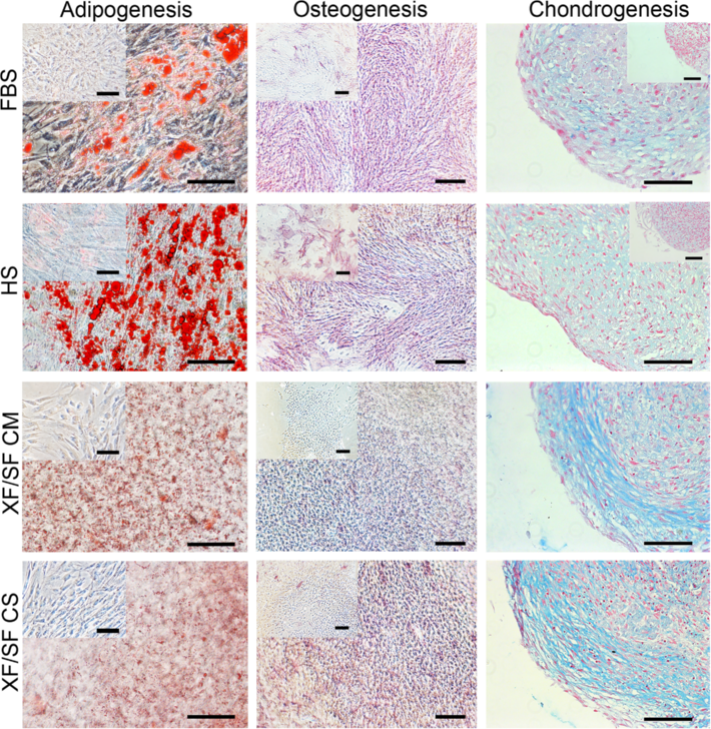
**Multilineage differentiation potential of ASCs.** Differentiation potential of ASCs cultured in four different conditions; FBS, HS, XF/SF medium with Coating Matrix Kit, or CELLstart coating was investigated toward adipogenic, osteogenic, and chondrogenic cells. Oil Red-O staining indicates the formation of intracellular lipid in cells going through adipogenic differentiation (scale bar, 100 μm); ALP staining reveals the alkaline phosphatase activity in osteogenic-differentiation cultures (scale bar, 300 μm), and Alcian blue staining recognizes the glycosaminoglycans of the cells going through chondrogenic differentiation (scale bar, 100 μm). Adipogenesis and osteogenesis was more effective in serum-containing media, whereas clearly the most intense chondrogenesis was seen in XF/SF cultures.

In the adipogenic-induction cultures, oil droplets were visible by light microscopy in cells expanded in HS- or FBScontaining medium. In XF/SF induction culture, differentiation was clearly initiated but did not progress very efficiently, which was shown by smaller oil droplets in Oil Red-O staining (Figure 5).

**Figure 6:**
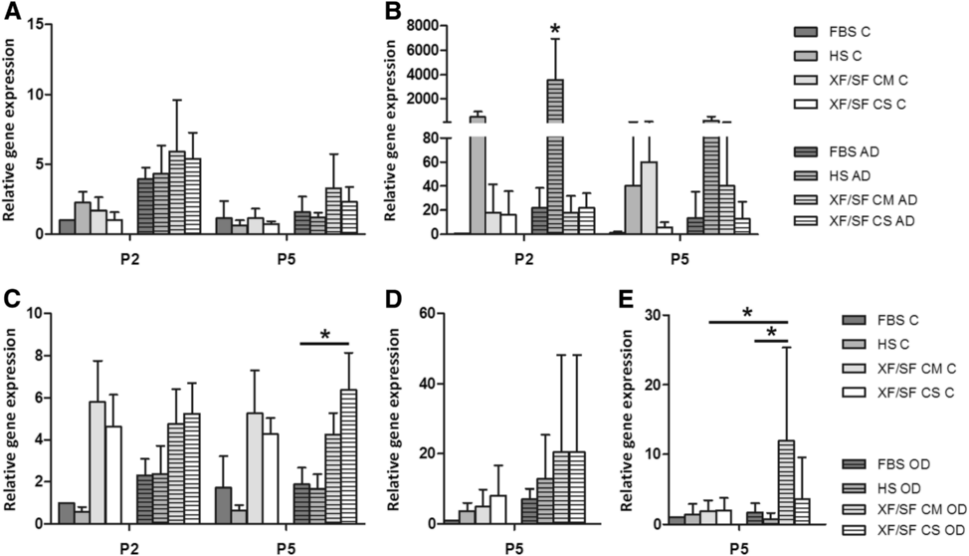
**Gene-expression analysis.** Differentiation-specific gene-expression analysis was performed after 14 days of differentiation induction, versus control medium, in different culture conditions: FBS, HS, XF/SF medium with Coating Matrix Kit, and with CELLstart coating in passages 2 and 5. The expression of *PPARγ* gene (**A**) and *aP2* gene (**B**) demonstrate adipogenesis after 14 days of adipogenic induction. Respectively, the expression of *Runx2* (**C**) *ALP* (**D**), and *DLX5* (**E**) genes indicate osteogenic differentiation after 14 days of osteogenic induction. The expression of *ALP* and *DLX5* are presented in passage 5. A significant increase in the expression of aP2 was observed in HS-supplemented induction medium compared with HS control medium, and the cells in all the other induction media in passage 2. Further, the expression of *Runx2* was significantly increased in XF/SF CM induction medium as compared with FBS/HS inductions in passage 5. Moreover, the response of *DLX5* to the osteogenic induction was significantly stronger in XF/SF CM-cultured cells when compared with FBS/HS conditions, as well as XF/SF CM control in passage 5. AD, adipogenic differentiation; OD, osteogenic differentiation. The data in the diagrams are presented as mean ± SD.

Nevertheless, a trend of higher expression of the gene *PPARγ*, the central transcriptional regulator of adipogenesis, was noted in XF/SF conditions as compared with serum-containing medium, but no significant differences were seen because of high standard deviation (Figure 6A). Further, the expression of *aP2* (fatty acid-binding protein) (Figure 6B) was consistent with the results of Oil Red-O staining (Figure 5). The most intense differentiation was seen in HS medium, which was demonstrated by large oil droplets in Oil Red-O staining and by a significant increase in the expression of *aP2* gene (Figure 6B) in ASCs cultured in HS-supplemented induction medium compared with HS control medium and the cells in all the other induction media in passage 2. Although the serum-containing medium and especially HS medium appeared to be the best condition for adipogenic differentiation, cells cultured under XF/SF conditions showed signs of early differentiation.

**Figure 7:**
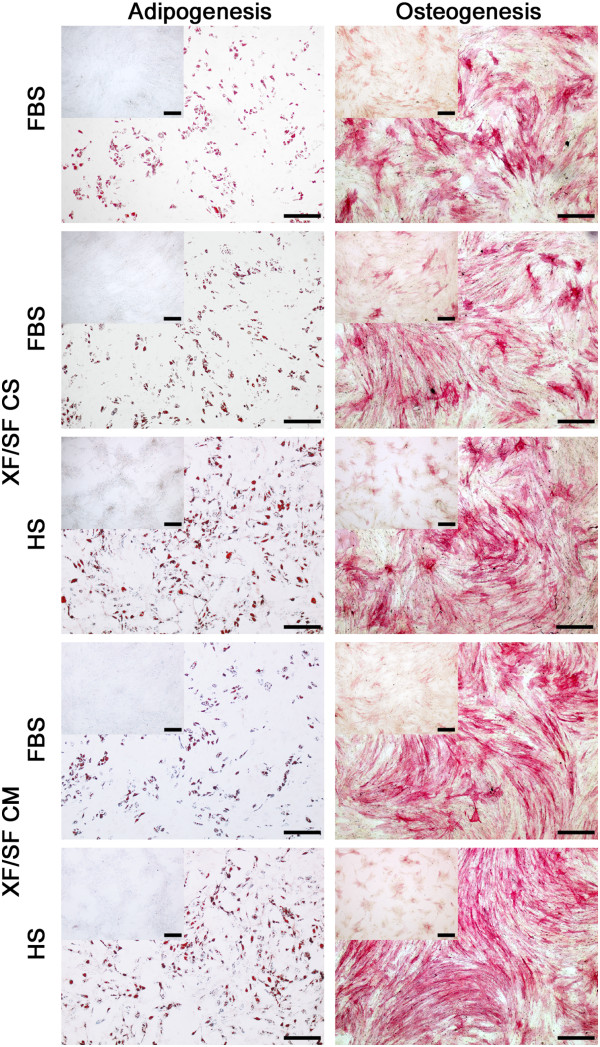
**Adipogenic and osteogenic differentiation potential of ASCs after serum priming.** Follow-up study on the differentiation of ASCs performed in five different conditions: (1) FBS, (2) XF/SF CS primed initially in FBS, (3) XF/SF CS primed initially in HS, (4) XF/SF CM primed initially in FBS, and (5) XF/SF CM primed initially in HS. Oil Red-O staining indicates the formation of intracellular lipid in cells going through adipogenic differentiation (scale bar, 100 μm); ALP staining reveals the alkaline phosphatase activity in osteogenic differentiation cultures (scale bar, 100 μm). Inset images are the paired undifferentiated negative controls. Differentiation toward adipogenic and osteogenic cells was more efficient when primed with HS- or FBS-containing medium.

Because the induction response of XF/SF cells to adipogenic differentiation appeared to be attenuated under serum-free condition, we hypothesized that the cells needed more nutrient-rich media to promote efficient adipogenesis. Cryopreserved ASCs cultured in FBS-containing media (passage 1), XF/SF CS (passage 2), and XF/SF CM (passage 2) were thawed, recovered, and grown in their own media. After reaching near confluency, the ASCs were harvested and plated into both FBS-containing or HS-containing media and let grow for 3 days. Then the medium was replaced with adipogenic induction media in either FBS- or HS-containing condition and cultured for 14 days. As predicted, differentiation was more efficient when primed with HS- or FBS-containing medium, but HS media clearly displayed increased differentiation than did FBS media (Figure 7). No noticeable differences were noted between XF/SF CS and XF/SF CM cells when induced with either FBS- or HS-based adipogenic media.

### Osteogenic differentiation

In the osteogenic induction cultures, cells expanded in HS- or FBS-containing medium showed slightly enhanced capacity to undergo osteogenic differentiation than did the cells expanded under XF/SF conditions, based on the ALP staining (Figure 5). However, the proliferation rate of ASCs grown in XF/SF medium was increased compared with serum-containing medium and, as a result, the wells became confluent, and cells started to detach. Therefore, the weak ALP staining in XF/SF conditions may indicate attachment difficulties of the cells in confluent wells, although the osteogenic differentiation is ongoing (Figure 5).

In addition to ALP staining, the osteogenic differentiation was evaluated by the expression of osteogenesis-specific genes. In contrast to ALP staining results, the expression of *Runx2* was increased in XF/SF conditions as compared with serum-containing medium, and a statistically significant increase was seen between XF/SF CM and FBS/HS induction media in passage 5 (Figure 6C). Further, the response of *DLX5* (Figure 6E) to the osteogenic induction was stronger in XF/SF cultured cells when compared with FBS/HS conditions, and a statistically significant increase in *DLX5* expression was seen in cells cultured in XF/SF CM induction media when compared with FBS/HS induction conditions, as well as XF/SF CM control. Also, the alkaline phosphatase staining result was verified at gene-expression level, where a trend of increased expression was observed in every differentiation culture condition when compared with control samples (Figure 6D). Although the expression of alkaline phosphatase (*ALP*) was increased after induction, no significant differences were seen because of high standard deviation.

To determine whether ALP staining could be enhanced, XF/SF cells were also tested with a serum-based medium to see whether more-efficient osteogenesis could be induced. Cryopreserved ASCs cultured under FBS-containing media (passage 1), XF/SF CS (passage 2), and XF/SF CM (passage 2) were thawed, recovered, and grown in their own media. After reaching near confluency, the ASCs were harvested and plated into both FBS- and HS-containing media and let grown for 3 days. After 3 days, the media were replaced with osteogenic induction medium in either FBS- or HS-containing condition and cultured for 14 days. Differentiation was more efficient when primed with HS- or FBS-containing medium (Figure 7). No noticeable differences were noted between XF/SF CS and XF/SF CM cells when induced with either FBS- or HS-based osteogenic media.

### Chondrogenic differentiation

Chondrogenic differentiation was more intense in XF/SF conditions compared with serum containing medium, shown by the Alcian blue staining of proteoglycans after the micromass culture in chondrogenic induction medium (Figure 5). The size of the pellet was also larger in XF/SF conditions versus HS or FBS cultures. These results suggest the XF/SF conditions promote the cartilage differentiation since the formation of proteoglycans, central components of cartilage tissue, is enhanced in XF/SF cultures.

## Discussion

Today, clinical cell therapies using ASCs are in progress, and several clinical trials are ongoing [5] and require more-reliable, reproducible, and safe methods for *in vitro* expansion of the cells. Therefore, the transition from FBS- or HS-medium supplementation to defined XF/SF culture conditions would be one of the most important steps forward in considering the suitability of ASCs for clinical use. By removing all the animal-derived components as well as the undefined serum from the cell-culture workflow, the safety of the patient receiving cell transplant can be improved.

Traditionally, ASC culture medium has been supplemented with FBS, which is not a preferred option in clinical therapies because of xenogeneic components with critical safety issues [12,25]. Consequently, different kinds of alternatives for FBS have been studied considering the clinical use of ASCs. Trivedi and colleagues [26] replaced FBS with 20% human albumin during the ASC expansion for clinical use to treat diabetes, whereas Tzouvelekis and colleagues [27] used autologous platelet-rich plasma for the cell expansion to treat patients with pulmonary fibrosis. AutoHS is currently used for the expansion of ASC by our group for the reconstruction of bone defects in the craniomaxillofacial area [4,28]. However, as mentioned earlier, limitations are associated with the use of autoHS or serum derivatives, such as lot-to-lot variability [17], limited availability, and undefined composition, and therefore, the use of XF/SF medium would be the preferred option.

Studies have been performed on ASCs/BMSCs studying the defined XF- or SF-culture conditions; Dromard and colleagues [29] demonstrated that ASCs can be expanded as floating spheres in defined SF-culture systems supplemented with 2% human plasma and specific growth factors. Further, Santos *et al*. [30] investigated a microcarrier-based bioreactor system for the XF/SF expansion of ASCs and BMSCs. Moreover, the suitability of human platelet lysate (PL) for FBS substitution has been investigated by several groups. Schallmoser and colleagues [15] introduced a standard protocol for platelet preparation for animal protein-free cultures of ASCs, and Naajikens *et al*. [31] showed that PL-cultured ASCs had a similar differentiation capacity and increased proliferation rate when compared with FBS cultures. Blande *et al.*[32], in contrast, showed that ASC population doubling time in PL cultures was significantly lower than that in FBS cultures, but the immunophenotype was similar, and both cultures retained the differentiation potential of the cells.

Still, a better-defined culture environment is needed, and to our knowledge, this study is the first report describing ASC isolation and expansion in completely XF/SF conditions maintaining the basic stem cell characteristics of ASCs. In the past, XF or SF expansion of mesenchymal stem cells has been reported, but the cell isolation and early expansion and differentiation studies were carried out in serum-containing medium [19,30,33-35]. In this study, we isolated the cells without serum exposure in XF/SF conditions by using BD PureCoat carboxyl flasks. Onward from passage 1, the cells were able to grow on basic Nunclon Δ surface vessels in XF/SF conditions when either CELLstart CTS coating or XF/SF CM was used. Considering the future clinical applications, it is an advance that the cells are isolated and expanded in fully XF/SF culture conditions instead of using serum supplementation at any point of the culture. If the patient is exposed to undefined components under *in vitro* expansion, an increased risk occurs for cross-contaminations and immune reactions in a patient receiving the cell transplant. Nevertheless, patient safety is still the most important aspect considering clinical use of ASCs.

In addition to safety, it is advantageous for *in vitro* expansion of cells to be performed in a shorter time scale. Our studies on the cell-proliferation rate were consistent with the results of earlier studies of mesenchymal stem cell cultures under XF or SF conditions [19,33,34] in which the higher proliferation rate of XF- or SF-cultured cells compared with FBS cultures was demonstrated. In our study, the cumulative population doubling in XF/SF medium was superior when compared with both FBS and HS conditions. Efficient expansion of ASCs in XF/SF conditions is crucial for clinical sustainability where a large cell number is required in a minimum time scale.

Cell surface-marker expression profile of ASCs was largely similar between cells grown under different culture conditions, except the significant difference in the expression of CD54 (ICAM-1), which is a marker of endothelial cells and cells of the immune system. To our knowledge, CD54 expression of ASCs has not been studied earlier in XF/SF conditions. The lower expression of CD54 may suggest that a more homogeneous cell population is achieved through more-selective isolation and expansion protocols compared with cells isolated in the presence of serum. In addition, weaker cell adhesion under XF/SF conditions was observed, which may reflect on lower expression of the adhesion molecule ICAM1 (CD54). This aspect of XF/SF cultures and the possible selective effect on cell population has to be investigated in subsequent XF/SF studies, especially when a coating is used.

Some variations were also seen in the expression of CD11a (integrand am), CD14 (lip polysaccharide receptor), CD19 (leukotriene B4 receptor), and CD86 (costimulatory molecule for T-cell activation) on cells grown in XF/SF conditions versus serum-containing medium. All of these markers are known to interact with immune-related cells, and therefore, the culture conditions may affect the immunogenicity of ASCs.

The minimal criterion for the immunophenotype of MSCs described by Dominici *et al*. [7] was defined for cells cultured under standard condition in a medium with FBS supplementation. However, our current results with XF/SF cells demonstrate that the cell surface-marker profile applies to ASCs cultured under XF/SF conditions as well. The hematopoietic progenitor cell marker CD34 was moderately expressed in both XF/SF and serum-supplemented conditions in contrast to earlier described criteria. However, similar results for CD34 expression have been reported by others [36,37], and the variable interpretations could be explained by differences cell-culturing and -passaging protocols. In the current study, the expression of CD34 and CD54 was higher in passage 2, but the expression level was decreased in later passages, indicating a more homogeneous population.

The chondrogenic differentiation capacity of ASCs cultured under XF/SF conditions was strong compared with cells cultured in serum-containing medium based on the Alcian blue staining. Similar findings have been shown by Chase *et al*. [31], in which a robust chondrogenesis in SF-culture conditions was seen when compared with serum-containing medium. In our study, the Alcian blue staining of proteoglycans was intense in cells expanded under XF/SF conditions, and also the pellet size after micromass-culture was larger compared with serum-containing medium, which was in agreement with Chase’s results. This result promises potential use of ASCs in chondrogenic applications, but further research is needed to investigate the chondrogenic-differentiation potential of ASCs in XF/SF conditions.

Moreover, the differentiation potential toward osteogenic and adipogenic cells was investigated in XF/SF conditions. Unlike in previous studies [28,31,32], osteogenesis and adipogenesis was induced in totally XF/SF differentiation media, and serum was substituted by XF/SF supplement of the STEMPRO MSC SFM kit. When ASCs were cultured under XF/SF conditions, they showed moderate differentiation potential toward osteogenic and adipogenic cells, as demonstrated by ALP and Oil Red-O staining, as the differentiation was not as efficient as seen in serum-containing medium. By optimizing the differentiation protocols for each condition, the efficiency of osteogenic and adipogenic differentiation can be enhanced. Furthermore, the reduced differentiation potential may be due to decreased cell adhesion for cells undergoing differentiation under XF/SF conditions. The weak cell-attachment hypothesis was supported by altered morphology and cell detachment during the proliferation studies, as well as decreased enzymatic digestion time. Another explanation is that ASCs need a more nutrient-rich media to promote robust differentiation. The follow-up differentiation studies with FBS- or HS-based media strongly indicate that nutrients play a key role in efficient differentiation. These findings are consistent with previous studies in which the importance of cell adhesion and nutrients during the cell differentiation has been shown [38,39].

In addition, the responses seem to be donor specific, and possibly, some cell lines respond better to the osteogenic induction, whereas others respond better to adipogenic induction. Thus, variation in the responses exists, and different stages of differentiation are evident, as shown by the high standard deviations of gene expressions. Also, the mRNA expression and enzymatic activity may not be in line because the regulation occur on posttranscriptional and translational levels, and finally, on the level of formation of an active enzyme. The cells cultured in different conditions may also be at different stages of their differentiation process, thus not expressing the same markers simultaneously. Still, the activity increase in gene-expression level shows commitment to osteogenic or adipogenic pathway, depending on donor cell line and culture condition.

Nevertheless, although at an early stage, differentiation occurred in XF/SF medium, showing that the cells have the capacity for trilineage differentiation, as shown by Oil Red-O, ALP, and Alcian blue staining. Chase *et al.*[33] demonstrated that BMSCs expanded in SF conditions and differentiated in serum containing induction medium retained their ability to differentiate into adipocytes, chondrocytes, and osteoblasts. In addition, Yang *et al.*[34] published similar results on the differentiation potential of ASCs expanded in a hypoxic XF environment. The cells expanded in XF medium had equal multilineage differentiation capacity, as compared with cells expanded in traditional serum-containing medium when serum induction was used during the differentiation. Taking these previous results into account, the efficiency of differentiation could easily be improved by serum induction during the culture in differentiation medium.

However, the aim of this study was to remove all the undefined components from the cell-culture workflow, and therefore, serum induction is not a preferred option for use during differentiation. Conversely, autoHS supplementation may be used in clinical treatments for differentiation induction, and the cells would still be expanded in defined XF/SF conditions before differentiation. Also, ASCs can be implanted to the defect site in their undifferentiated state, and the cell differentiation then occurs *in vivo,* as has been performed by our group [4,28].

Additionally, growth factors and biomaterials can be used to support cell differentiation in XF/SF conditions. Cordonnier and co-workers [40] showed that growth-factor induction is especially effective for cells cultured in low serum. In their study, the effect of bone morphogenetic proteins (BMPs) -2, -4, and −7 on osteogenic differentiation of BMSCs was evaluated in low (2%) and higher (10%) FBS-supplemented cultures, leading to a conclusion that BMP-4 induction in low-serum cultured cells was the most effective osteogenic inducer. Similarly, the osteogenic differentiation potential of XF/SF cultured ASCs could be enhanced by growth-factor induction. Furthermore, the differentiation capacity of XF/SF-cultured ASCs can be enhanced by inductive biomaterials, such as osteopromoting bioactive glass [41] or β-tricalcium phosphate [42], or alternatively, chondrogenesis-inductive materials such as 3-D woven polycaprolactone scaffolds [43].

## Conclusions

Effective and safe *in vitro* methods to isolate and expand ASCs are critical for the positive development of cell-therapy applications. Our current results demonstrate that the novel XF/SF culture conditions maintains the stem cell characteristics of ASCs. The cells grown in different culture conditions displayed the characteristic immunophenotype of ASCs with minor differences. Importantly, the proliferation rate of ASCs was significantly increased in XF/SF conditions, compared with HS- and FBS-containing medium. Furthermore, the chondrogenic differentiation potential was intense in XF/SF conditions, whereas adipogenic and osteogenic differentiation were comparable to the FBS condition after serum priming.

These novel XF/SF culture conditions have great potential for clinical use, but additional preclinical safety and efficacy studies will be needed and standardized before using in clinical treatments. Off-the-shelf cell products will require effective XF/SF conditions in which the basic stem-cell characteristics of ASCs are maintained, the proliferation rate is high, and the cells retain their functionality. Naturally, a substantial number of safety-assessment studies would have to be done before allogeneic ASCs can be used in clinical cell treatments. The development of efficient and safe XF/SF-culture conditions is one step closer to that goal.

## Abbreviations

AD: Adipogenic differentiation; alloHS: Allogeneic human serum; ALP: Alkaline phosphatase; aP2: Adipocyte protein 2; ASC: adipose stem cells; autoHS: Autologous human serum; BMPs: Bone morphogenic proteins; BMSC: Bone marrow-derived stem cell; CD: Chondrogenic differentiation; CM: Coating matrix; CS: CELLStart; DLX5: Distal-less homeobox transcription factor 5; FBS: Fetal bovine serum; HS: human serum; ICAM1: Intercellular adhesion molecule 1; MSC: mesenchymal stem cell; OD: Osteogenic differentiation; PPARγ: peroxisome proliferator-activated receptor γ; RUNX2: runt-related transcription factor 2; XF/SF: Xeno- and serum-free.

## Competing interests

SB, AC, and MV are regular employees of Life Technologies and have not received any financial gains. They hold some stocks of Life Tech as employees of Life Technologies. MP, MJ, BM, and SM declare that they have no competing interests. The authors alone are responsible for the content and writing of the manuscript.

## Authors’ contributions

MP performed the laboratory work, the isolation and expansion of adipose stem cells, proliferation, immunophenotypic, and differentiation studies in cooperation with MJ. MP performed statistical analyses and wrote the manuscript, and MJ participated in producing the figure panels and reviewing the manuscript. BM designed and supervised the study and participated in reviewing the manuscript. SB participated in designing the study, performed the follow-up differentiation studies, and reviewed the manuscript. SM supervised the study and participated in reviewing the manuscript. AC participated in the development of XF/SF coating-free supplements and reviewed the manuscript. MV participated in planning and reviewing of the results and discussion. All authors read and approved the final manuscript.
